# The Diagnostic Gap Between Clinical and Pathological Extranodal Extension in Head and Neck Cancers: A 5-Year Nationwide Trend Analysis in Taiwan

**DOI:** 10.3390/jpm16020123

**Published:** 2026-02-20

**Authors:** Hsuen-Fu Lin, Shih-Han Hung

**Affiliations:** 1 Department of Hematology and Oncology, Mennonite Christian Hospital, Hualien 970, Taiwan; mosqlin@gmail.com; 2 Department of Otolaryngology-Head and Neck Surgery, Mennonite Christian Hospital, Hualien 970, Taiwan

**Keywords:** extranodal extension, head and neck cancer, diagnostic gap, Taiwan Cancer Registry, AJCC eighth edition, personalized medicine

## Abstract

**Background:** Extranodal extension (ENE) is a critical prognostic factor in head and neck squamous cell carcinoma (HNSCC) and was incorporated into the AJCC eighth-edition staging system. However, the concordance between clinical (cENE) and pathological (pENE) ENE remains poorly understood in real-world practice. **Methods:** We conducted a retrospective analysis using Taiwan Cancer Registry (TCR) long-form data from 2018 to 2022, focusing on four major HNSCC sites (oral cavity, oropharynx, hypopharynx, and larynx). The diagnostic gap was defined as the difference between pENE and cENE positivity rates. **Results:** Among 29,830 patients, a persistent diagnostic gap was observed across all sites: laryngeal (20.8%), hypopharyngeal (20.4%), oropharyngeal (11.5%), and oral cavity (9.9%). For oral cavity cancer, the gap did not narrow over the 5-year period (*p* = 0.9788). Furthermore, in oral cavity cancer, medical centers demonstrated a larger gap than non-medical centers (10.5% vs. 8.4%), a phenomenon we term the “Quality-Gap Paradox”. **Conclusions:** A significant diagnostic gap persists in HNSCC, highlighting the limitations of current imaging. The Quality-Gap Paradox, observed in oral cavity cancer, suggests this is driven by a complex interplay of factors including superior pathological detection in high-volume centers. Our findings underscore the need for advanced, personalized risk-stratification tools to bridge this gap and improve patient management.

## 1. Introduction

Extranodal extension (ENE), the invasion of cancer cells through the lymph node capsule into surrounding connective tissue, is one of the most powerful adverse prognostic factors in head and neck squamous cell carcinoma (HNSCC) [1,2]. Its presence is associated with a significant increase in the risk of distant metastasis and a decrease in overall survival, often halving a patient’s prognosis [3]. Recognizing its profound impact, the American Joint Committee on Cancer (AJCC) incorporated ENE into the eighth-edition staging system for HNSCC, upstaging any ENE-positive node to the N3b category, regardless of size or number [4].

However, the clinical implementation of this staging criterion is hampered by a significant challenge: the diagnostic gap between clinical ENE (cENE), detected preoperatively by imaging modalities like computed tomography (CT) and magnetic resonance imaging (MRI), and pathological ENE (pENE), the gold standard confirmed by postoperative histopathological examination. Systematic reviews have consistently shown that the sensitivity of imaging for detecting ENE is suboptimal, often hovering around 60–70% [5,6]. This means a substantial proportion of patients with microscopic ENE (mENE) are not identified before surgery, leading to potential under-staging and under-treatment.

This issue is particularly relevant in Taiwan, where oral cavity cancer is a major public health concern, largely driven by the widespread practice of betel quid chewing [7,8]. The high incidence provides a unique opportunity to study the real-world implications of the AJCC eighth edition on a large, nationwide scale. To our knowledge, no large-scale study has evaluated the trend of the cENE-pENE diagnostic gap in Taiwan since the implementation of the new staging system. Understanding the magnitude, trend, and contributing factors of this gap is crucial for improving diagnostic accuracy, refining treatment strategies, and advancing personalized medicine in HNSCC.

This study aims to conduct a comprehensive, nationwide analysis of the diagnostic gap between cENE and pENE in four major HNSCC sites (oral cavity, oropharynx, hypopharynx, and larynx) using data from the Taiwan Cancer Registry (TCR) from 2018 to 2022. We hypothesize that a significant diagnostic gap persists and has not narrowed over time. We will also investigate the “Quality-Gap Paradox”—the counterintuitive hypothesis that higher-quality medical centers may exhibit a larger gap due to more meticulous pathological examination.

## 2. Materials and Methods

### 2.1. Data Source and Study Population

This retrospective cohort study utilized data from the Taiwan Cancer Registry (TCR) long-form database, a mandatory, population-based cancer registry covering over 98% of all cancer cases in Taiwan. We included all patients newly diagnosed with squamous cell carcinoma of the oral cavity, oropharynx, hypopharynx, and larynx between 1 January 2018, and 31 December 2022. This period was chosen to coincide with the implementation of the AJCC 8th edition, ensuring consistent ENE definitions. Patients with distant metastasis at diagnosis (M1), unknown ENE status, or those who did not undergo neck dissection were excluded from the pENE analysis.

### 2.2. Variable Definitions

Site-Specific Factors (SSF) in the TCR are standardized codes used to capture detailed, cancer-specific information not included in general staging. For HNSCC, SSF9 and SSF10 are used to document ENE status.

Clinical ENE (cENE): Defined based on SSF9. A case was considered cENE-positive if the code was 010, 020, or 030. These codes correspond to clinical or radiographic evidence of ENE based on imaging findings such as ill-defined nodal borders, infiltration into adjacent fat or muscle, or frank invasion of nearby structures, consistent with recently standardized international criteria [9,10]. The cENE rate was the number of cENE-positive cases divided by the total number of cases with a valid SSF9 entry.

Pathological ENE (pENE): Defined based on SSF10. A case was considered pENE-positive if the code was 010, 020, or 030. These codes indicate pathologically confirmed ENE, defined as tumor extension through the lymph node capsule into the surrounding perinodal soft tissue, as identified by histopathological examination. The pENE rate was the number of pENE-positive cases divided by the total number of cases that underwent neck dissection.

Hospital Level: In Taiwan, hospitals are officially accredited by the Ministry of Health and Welfare into different tiers. Medical centers represent the highest tier, equivalent to tertiary care or academic medical centers in other healthcare systems. These institutions are required to meet stringent criteria, including: (1) provision of comprehensive subspecialty services across all major disciplines; (2) maintenance of advanced diagnostic and therapeutic technologies; (3) active involvement in medical education and training programs; and (4) demonstrated capacity for high-volume, complex case management. Non-medical centers include regional hospitals (comparable to secondary care hospitals) and district hospitals, which provide general and some specialized services but may not have the full range of subspecialties or the same research and teaching infrastructure. For this study, hospitals were categorized as medical centers or non-medical centers based on the official accreditation status provided by the TCR at the time of patient diagnosis.

Diagnostic Gap: The primary outcome, calculated as the absolute difference between the pENE rate and the cENE rate (Gap = pENE% − cENE%). It is important to note that the diagnostic gap, as defined here, is a population-level metric that quantifies the overall under-detection of ENE by clinical/imaging assessment relative to pathological examination. It does not represent individual-patient concordance or discordance, nor does it directly measure the sensitivity or specificity of clinical ENE detection, which would require patient-level paired cENE and pENE data. Rather, the gap reflects the aggregate difference in detection rates across the population and serves as an indicator of the clinical challenge in preoperatively identifying ENE.

### 2.3. Statistical Analysis

Descriptive statistics were used to summarize patient characteristics and ENE rates. The Cochran-Armitage test for trend was used to evaluate the 5-year trend of cENE and pENE rates for oral cavity cancer. Chi-square tests were used to compare ENE rates between hospital levels and across different cancer sites. Pairwise comparisons of pENE rates across the four HNSCC sites were performed using Chi-square tests with statistical significance set at *p* < 0.05. All statistical analyses were performed using R software (version 4.2.1). A *p*-value of <0.05 was considered statistically significant.

### 2.4. Limitations of Hospital-Level Comparison

For the hospital-level comparison (medical centers vs. non-medical centers), we acknowledge that these groups may differ in case mix, tumor stage distribution, and referral patterns. Medical centers, as tertiary care institutions, often receive referrals of more advanced or complex cases, which could confound the observed differences in diagnostic gap. Ideally, stage-adjusted or subsite-specific sensitivity analyses would be performed to account for these differences. However, the TCR long-form data structure does not provide sufficient granularity for such stratified analyses at the hospital level. Specifically, the registry does not include detailed T-stage, N-stage, or anatomical subsite information cross-tabulated with hospital-level data in a format that would allow for robust adjusted analyses. Therefore, our hospital-level findings should be interpreted as hypothesis-generating, and residual confounding by case complexity cannot be entirely ruled out. Future studies with more detailed patient-level data would be needed to confirm whether the Quality-Gap Paradox persists after adjustment for stage and other clinical factors.

### 2.5. Declaration of AI Tool Usage

AI-assisted technologies were used strictly for editorial purposes during the manuscript preparation. The WPS Office AI tool was utilized to improve English writing fluency, and the Perplexity platform assisted in checking citation accuracy. The authors reviewed all AI-suggested changes and maintain full responsibility for the integrity and originality of the work.

## 3. Results

### 3.1. Patient Characteristics

A total of 29,830 patients with newly diagnosed HNSCC from 2018 to 2022 were included in the analysis. Oral cavity cancer was the most common site (n = 27,815), followed by oropharynx, hypopharynx, and larynx. The majority of patients were male, with a median age in the late 50s.

### 3.2. Diagnostic Gap Analysis

**Figure 1:**
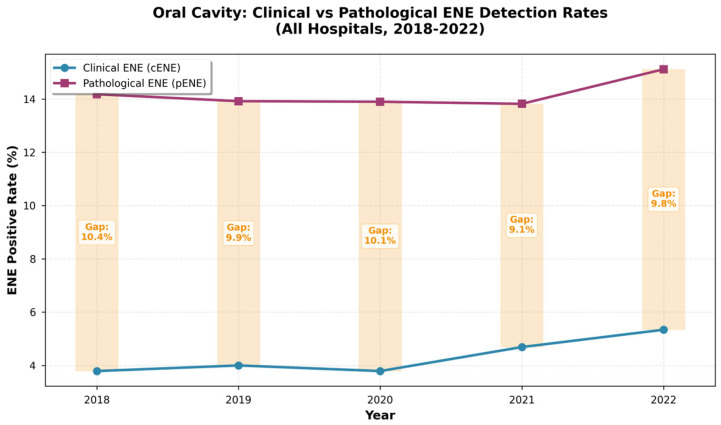
Five-year trend of clinical extranodal extension (cENE) and pathological extranodal extension (pENE) rates in oral cavity cancer patients in Taiwan (2018–2022). The diagnostic gap (pENE rate − cENE rate) remained stable over the study period (Cochran-Armitage trend test, *p* = 0.9788).

Across all sites, a significant and persistent diagnostic gap was observed. For oral cavity cancer, the 5-year average cENE rate was 3.8%, while the pENE rate was 13.7%, resulting in an average diagnostic gap of 9.9%. The trend of cENE and pENE rates over the five years is illustrated in Figure 1. The Cochran-Armitage test for trend showed no significant change in the diagnostic gap over the 5-year period (*p* = 0.9788), indicating that the gap did not narrow despite increased awareness following the AJCC eighth edition implementation.

### 3.3. Comparison Across Cancer Sites

**Figure 2:**
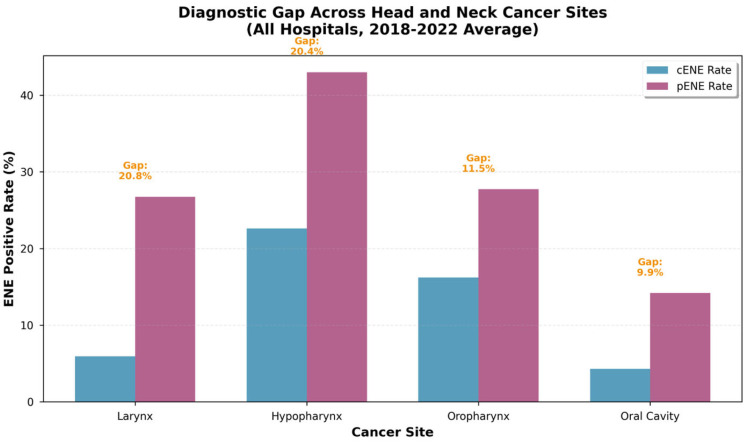
Comparison of the 5-year average diagnostic gap across four major head and neck cancer sites. Laryngeal and hypopharyngeal cancers demonstrated the largest gaps, followed by oropharyngeal and oral cavity cancers.

A comparison of the 5-year average diagnostic gap across the four major HNSCC sites is shown in Figure 2. Laryngeal cancer exhibited the largest gap (20.8%), followed by hypopharyngeal cancer (20.4%), oropharyngeal cancer (11.5%), and oral cavity cancer (9.9%). Chi-square tests revealed that these differences in pENE rates were statistically significant for most pairwise comparisons (all *p* < 0.001), with the exception of oropharynx versus larynx (*p* = 0.74). Notably, both laryngeal and hypopharyngeal cancers demonstrated gaps approximately twice as large as oral cavity cancer, suggesting site-specific factors influence the diagnostic accuracy of ENE detection.

### 3.4. The Quality-Gap Paradox

**Figure 3:**
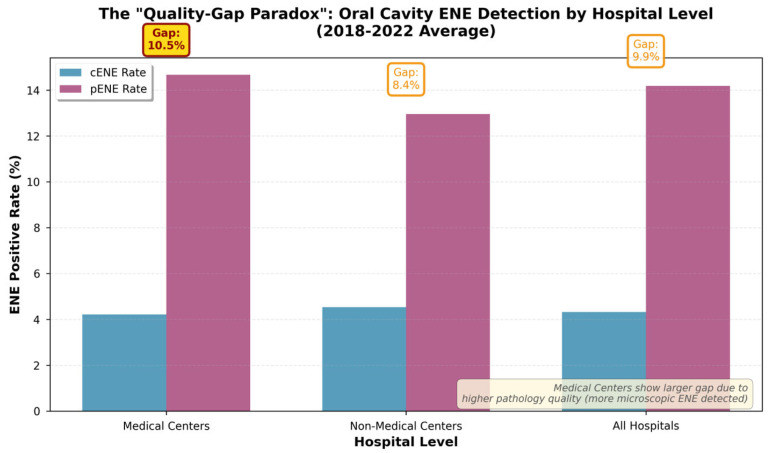
The “Quality-Gap Paradox” in oral cavity cancer. The 5-year average diagnostic gap was significantly larger in medical centers compared to non-medical centers, reflecting higher pathological detection rates.

When stratified by hospital level, a paradoxical finding emerged. For oral cavity cancer, medical centers had a significantly larger 5-year average diagnostic gap (10.5%) compared to non-medical centers (8.4%). This difference was primarily driven by a higher pENE detection rate in medical centers (14.7% vs. 13.0%), while cENE rates were similar (4.2% vs. 4.6%). Chi-square analysis confirmed that the difference in pENE rates between medical centers and non-medical centers was statistically significant (χ^2^ = 8.73, *p* = 0.003), indicating that the observed paradox is not due to chance. This paradox is visualized in Figure 3, which compares the diagnostic gap between the two hospital levels.

## 4. Discussion

This is the first nationwide study in Taiwan to comprehensively evaluate the diagnostic gap between clinical and pathological extranodal extension (ENE) across four major head and neck cancer sites since the implementation of the American Joint Committee on Cancer (AJCC) eighth edition staging system. Our analysis of over 29,000 HNSCC cases from 2018 to 2022 reveals three principal findings with profound implications for personalized medicine. First, a significant and persistent diagnostic gap of approximately 10–20% exists, indicating a substantial rate of underdiagnosis of ENE by preoperative imaging. Second, this gap did not narrow over the five-year study period, suggesting that the introduction of ENE as a formal staging criterion has not, by itself, improved the clinical detection rate. Third, and most intriguingly, we identified a “Quality-Gap Paradox,” wherein medical centers, despite presumably having more advanced imaging and expertise, exhibited a larger diagnostic gap than non-medical centers. This suggests the gap is driven more by the quality of pathological examination than by deficiencies in clinical assessment. These findings challenge the current “one-size-fits-all” staging paradigm and underscore the urgent need for personalized, risk-adapted treatment strategies in HNSCC.

### 4.1. The Unwavering Diagnostic Gap: A Barrier to Personalized Risk Stratification

The persistence of the diagnostic gap is a stark reminder of the inherent limitations of current standard-of-care imaging modalities. Our finding of a ~10% gap in oral cavity cancer aligns with a growing body of literature documenting the challenge of accurately detecting ENE preoperatively [5,6]. The core of the issue lies in the distinction between macroscopic ENE, which is often visible on imaging, and microscopic ENE (mENE), where tumor cells breach the lymph node capsule on a microscopic level without causing significant morphological changes [9]. Current imaging techniques are simply not equipped to resolve these microscopic invasions.

From a personalized medicine perspective, this diagnostic gap represents a critical failure in patient stratification. It challenges the current “one-size-fits-all” staging paradigm, where all patients within a given AJCC stage are often considered for similar treatment pathways, despite underlying biological heterogeneity. The inability to preoperatively identify the 10–20% of patients with occult ENE has direct consequences for treatment personalization. The presence of pENE is a major indication for adjuvant therapy intensification. A patient correctly identified with ENE preoperatively might be considered for neoadjuvant systemic therapy, whereas a patient only found to have pENE postoperatively is typically recommended for adjuvant chemoradiation rather than radiation alone. This failure to identify high-risk patients upfront prevents the delivery of truly personalized, risk-adapted care from the outset and subjects patients to a treatment plan based on an incomplete assessment of their disease.

In 2024, the Head and Neck Cancer International Group (HNCIG) published consensus guidelines to standardize the radiological diagnosis of ENE (rENE) [10]. However, these criteria are still based on morphological changes often absent in mENE. Our data, showing no improvement in the gap from 2018 to 2022, suggests that even with heightened awareness, clinicians are hitting a ceiling in diagnostic capability with the tools at hand. This underscores the urgent need for novel technologies that can non-invasively detect microscopic disease, moving beyond anatomical imaging to functional or molecular characterization—a cornerstone of modern personalized oncology.

The lack of improvement in the diagnostic gap over the 5-year study period (*p* = 0.9788 for oral cavity cancer) is particularly noteworthy and warrants careful interpretation. Several factors may explain this stagnation. First, while the AJCC eighth edition heightened awareness of ENE as a critical staging parameter, the fundamental imaging technology (CT and MRI) has not undergone revolutionary advances in spatial resolution during this period. The physical limitations of current imaging modalities in detecting microscopic disease remain unchanged. Second, international standardized criteria for radiological ENE diagnosis were only published in 2024 [10], after our study period, suggesting that earlier years may have suffered from inconsistent interpretation and a lack of consensus on diagnostic thresholds. Third, it is plausible that pathological detection has also improved in parallel with clinical awareness, as pathologists became more vigilant in examining lymph nodes for ENE following the AJCC eighth edition’s emphasis on this feature. If both clinical and pathological detection improved proportionally, the gap would remain constant even as absolute detection rates increased.

The variation in diagnostic gap across different HNSCC sites is also highly informative and reflects the complex interplay of anatomical, biological, and technical factors. Laryngeal (20.8%) and hypopharyngeal (20.4%) cancers exhibited gaps approximately twice as large as oral cavity cancer (9.9%), and these differences were statistically significant (*p* < 0.001). This disparity likely reflects anatomical and technical factors. Oral cavity tumors are more accessible to direct clinical examination and high-resolution imaging, whereas laryngeal and hypopharyngeal lesions are located in deeper, more complex anatomical regions where lymph nodes may be obscured by surrounding structures, cartilage, and air-tissue interfaces, reducing imaging sensitivity. Furthermore, the lymphatic drainage patterns differ, with laryngeal and hypopharyngeal cancers more frequently involving deep cervical nodes that are challenging to assess radiologically. Interestingly, oropharyngeal and laryngeal cancers had similar gaps (*p* = 0.74), despite differences in anatomical location, suggesting that other factors such as tumor biology or nodal involvement patterns may also play a role.

### 4.2. The Quality-Gap Paradox: A Multifactorial Hypothesis

Perhaps the most significant finding of our study is the “Quality-Gap Paradox.” The observation that medical centers have a consistently larger diagnostic gap in oral cavity cancer (10.46% vs. 8.42%) is statistically significant (χ^2^ = 8.73, *p* = 0.003) and, on the surface, counterintuitive. While one might expect superior imaging at these centers to yield a smaller gap, the opposite finding suggests a complex interplay of factors. We propose this paradox is not attributable to a single cause but is likely a multifactorial phenomenon driven by differences in pathological, surgical, and radiological practices.

One primary hypothesis remains the role of pathological scrutiny. Medical centers are more likely to have dedicated head and neck subspecialty pathologists who may perform more meticulous lymph node examination, thereby identifying a higher rate of true pENE, particularly microscopic ENE (mENE), which was missed on imaging [9]. From this perspective, a larger gap could reflect a more thorough and accurate pathological workup, which is crucial for guiding appropriate adjuvant therapy. However, as noted by experts, pathologist skill is not exclusively confined to large centers, and expert practitioners may exist in any setting. Therefore, other factors must be considered.

Alternative or contributing factors may include surgical and radiological procedures. For instance, surgeons at high-volume medical centers might perform more extensive or comprehensive neck dissections, retrieving a higher number of lymph nodes for examination and thus increasing the probability of detecting occult pENE. Furthermore, while medical centers may have advanced imaging technology, the radiological diagnostic criteria for ENE were not internationally standardized until recently [10], potentially leading to variations in interpretation that are independent of hospital level. The observed gap may therefore reflect a combination of more comprehensive surgery and more detailed pathological analysis in medical centers, rather than a simple deficiency in any single area. This reframes the diagnostic gap as an indicator of system-level differences in the cancer care pathway, highlighting the need for further research to dissect the relative contributions of each component. Ultimately, this discussion should not be interpreted as a critique of non-medical centers, but rather as a hypothesis-generating observation that the drivers of staging discordance are complex and multifactorial. The study by Hondorp et al. on clinical-pathologic stage discordance similarly found only moderate agreement, emphasizing that the final pathologic stage remains the gold standard for a reason [11]. Our paradox provides a real-world, population-level validation of this principle and suggests that “personalized pathology,” characterized by expert, meticulous examination, is as important as personalized imaging or therapy.

An important caveat to our hospital-level analysis is the potential for residual confounding. Medical centers, as tertiary referral institutions, may treat a higher proportion of advanced-stage or complex cases compared to non-medical centers. If medical centers see more patients with advanced nodal disease, this could contribute to a higher pENE detection rate independent of pathological scrutiny. We were unable to perform stage-adjusted analyses due to limitations in the granularity of the TCR data at the hospital level. Therefore, while our findings are suggestive and hypothesis-generating, definitive conclusions about the drivers of the Quality-Gap Paradox will require future studies with patient-level data that allow for adjustment of tumor stage, nodal burden, and other clinical factors.

### 4.3. Future Directions: The Potential of AI in Bridging the Gap

Our findings highlight the urgent clinical need for advanced tools to bridge the diagnostic gap and improve patient stratification. The diagnostic impasse revealed by our study sets the stage for integrating artificial intelligence (AI) as a potential future solution. The limitations of human interpretation of complex imaging data are precisely the kind of problem that deep learning is poised to solve. Recent landmark studies have shown that AI models can not only predict ENE from CT scans but also quantify the burden of AI-predicted ENE, offering a powerful new prognostic tool [12,13]. This technology holds the promise of moving from a binary clinical assessment to a continuous, quantitative risk score, allowing for truly personalized treatment planning before the first incision is made. While our study did not involve AI analysis, it underscores the necessity of exploring and validating these technologies in future research.

### 4.4. Clinical Implications and the Path to Implementation

The clinical implications of our findings are immediate and actionable. First, clinicians must maintain a high index of suspicion for occult ENE, even when imaging is negative, particularly in high-risk scenarios (e.g., multiple involved nodes, large nodal size). Second, the “Quality-Gap Paradox” strongly advocates for the centralization of head and neck cancer care, particularly pathology services, to ensure all patients benefit from expert, high-quality diagnosis. Third, our study provides a clear mandate for the clinical validation and adoption of AI-based tools. Healthcare systems and professional societies should invest in the infrastructure and training needed to integrate these technologies into routine clinical workflows.

### 4.5. Strengths and Limitations

The primary strength of our study is its large, population-based design, which provides a real-world snapshot of the diagnostic gap across an entire country. However, some limitations must be acknowledged. First, the TCR database lacks detailed data on specific imaging protocols or radiologist experience, which could influence cENE detection. Second, we could not correlate findings with patient outcomes, as survival data for this recent cohort is not yet mature. Future studies should link these findings to survival to quantify the prognostic impact of the diagnostic gap. Third, our diagnostic gap metric is a population-level measure and does not assess individual-patient concordance between cENE and pENE. We did not calculate sensitivity, specificity, positive predictive value, or negative predictive value of clinical ENE detection, as this would require patient-level paired data linking each patient’s cENE status to their pENE status. The TCR long-form data structure does not provide such paired information at the individual level. Therefore, our findings should be interpreted as reflecting overall under-detection rates rather than diagnostic accuracy metrics. Future studies with access to individual patient records could provide complementary insights into the diagnostic performance of imaging at the patient level. Fourth, an inherent limitation of our study design is that pENE data are available only for patients who underwent neck dissection, while cENE data include all patients with imaging assessment. This creates a selection bias, as patients undergoing neck dissection are typically those with clinically or radiologically suspected nodal disease, representing a higher-risk subset of the overall HNSCC population. Consequently, the observed pENE rates may be higher than they would be if all patients underwent surgical pathological examination, and the diagnostic gap may be artificially widened. However, this limitation is unavoidable in real-world registry data, as early-stage patients without clinical nodal involvement do not undergo neck dissection as part of standard care. Despite this, our findings remain clinically meaningful because they reflect the actual diagnostic challenge faced by clinicians: among patients who do undergo surgery and for whom accurate preoperative staging is most critical, imaging still systematically under-detects ENE.

## 5. Conclusions

In conclusion, our nationwide study reveals a significant and persistent diagnostic gap between cENE and pENE in Taiwan following the implementation of the AJCC eighth edition. This gap, which did not narrow over a five-year period, underscores the inherent limitations of current imaging modalities in detecting microscopic ENE. The existence of a “Quality-Gap Paradox,” where medical centers exhibit a larger gap, highlights the crucial role of high-quality pathological examination in accurate staging. These findings challenge the current staging paradigm and signal an urgent need to move from population-based staging to individualized risk assessment. The integration of AI-powered predictive tools, alongside traditional clinical and pathological evaluation, represents the future of personalized HNSCC management, promising to bridge the diagnostic gap and tailor treatment strategies to each patient’s unique disease biology.

## Data Availability

The data presented in this study are available on request from the Taiwan Cancer Registry. Restrictions apply to the availability of these data, which were used under license for the current study, and so are not publicly available. The aggregated data analyzed are presented within the manuscript.
